# A large deletion encompassing exon 2 of the *ectodysplasin A* (*EDA*) gene in a British blue crossbred calf with hypohidrotic ectodermal dysplasia

**DOI:** 10.1186/s13028-022-00641-2

**Published:** 2022-09-06

**Authors:** Giovanni Capuzzello, Joana Gonçalves Pontes Jacinto, Irene Monika Häfliger, Gail E. Chapman, Sara Soto Martin, Lorenzo Viora, Nicholas N. Jonsson, Cord Drögemüller

**Affiliations:** 1 College of Medical, Veterinary & Life Sciences, University of Glasgow, 464 Bearsden Rd, Glasgow, G61 1QH UK; 2 Department of Veterinary Medical Sciences, University of Bologna, Via Tolara di Sopra 50Ozzano dell’Emilia, 40064 Bologna, Italy; 3 Institute of Genetics, Vetsuisse Faculty, University of Bern, 3012 Bern, Switzerland; 4 Institute of Animal Pathology, Vetsuisse Faculty, University of Bern, 3012 Bern, Switzerland

**Keywords:** Anodontia, Anomaly, Cattle, Development, Genodermatosis, Hypotrichosis, Precision medicine, Rare disease, Whole-genome sequencing

## Abstract

**Background:**

Hypohidrotic ectodermal dysplasia (HED) is a congenital syndrome of mammals affecting organs and tissues of ectodermal origin characterized by absence or hypoplasia of hair, teeth, and eccrine glands. The disorder has been reported in several species, including humans, mice, dogs and cattle, associated with variants in genes affecting the ectodysplasin pathway, including the X-linked ectodysplasin A (*EDA*) gene. Until now, nine pathogenic variants have been found in the bovine *EDA* gene. Here we report a novel variant in *EDA* in a crossbreed male Belgian Blue calf with HED, and provide an overview of the phenotypic and allelic heterogeneity of *EDA*-related forms of HED in cattle.

**Case presentation:**

A 45-day-old male crossbreed British Blue calf was referred with congenital hypotrichosis, oligodontia and omphalitis. On histopathological examination of the nasal planum, nasolabial glands and ducts were not observed. The density of hair follicles was low, and they were small, with a predominance of telogen-phase hairs, and some serocellular crusts. The phenotype of the calf resembled that of HED. Whole-genome sequencing (WGS) was performed and revealed a 21,899 base-pair deletion encompassing the coding exon 2 of *EDA,* predicted to result in an altered transcript and aberrant protein.

**Conclusions:**

The clinicopathological and genetic findings were consistent with a case of X-linked HED. A very similar *EDA* deletion has been previously reported in a family of Holstein cattle with HED. The newly identified hemizygous *EDA* loss-of-function variant is certainly pathogenic and therefore is the genetic cause for the observed phenotype. This case report provides an additional example of the potential of WGS-based precise diagnostics in livestock species such as cattle to increase the diagnostic yield in rare diseases.

**Supplementary Information:**

The online version contains supplementary material available at 10.1186/s13028-022-00641-2.

## Background

Ectodermal dysplasias are a heterogeneous group of rare genetic disorders characterized by impaired development of hair, teeth, and eccrine glands in mammals [1]. Hypohidrotic ectodermal dysplasia (HED) is an inherited genodermatosis described in various species including cattle [2], dogs [3], mice [4] and humans [5, 6]. Variants in any of the three ectodysplasin pathway genes, *ectodysplasin A* (*EDA*), *ectodysplasin A receptor* (*EDAR*), and *EDAR associated death domain* (*EDARADD*)*,* which encode a ligand, a receptor, and an intracellular signal mediator respectively of a single linear pathway that is important for the development of ectodermal appendages, are often causative of the disease [7, 8]. The main clinicopathological findings are the absence or hypoplasia of hair, teeth and eccrine glands. In cattle, this disorder is considered to be sub-lethal and has been associated with variants in *EDA* (OMIA 000543–9913) and *EDAR* (OMIA 002128–9913) genes.

The purpose of this study was to report a new variant in the *EDA* gene leading to HED and provide an overview of the phenotypic and allelic heterogeneity of *EDA*-related ectodermal dysplasia in cattle.

## Case presentation

A male British blue cross calf was referred to the Scottish Centre for Production Animal Health and Food Safety (SCPAHFS) at the University of Glasgow, School of Veterinary Medicine due to its abnormally short and fine hair, and an infection of the lower respiratory tract. The calf was born on a bovine viral diarrhoea virus (BVDV)-free dairy farm to a 2-year-old Holstein–Friesian dairy heifer that had been artificially bred to a British blue sire. The calf was born without assistance three weeks (258 days of pregnancy) before the due date (average length of gestation Holstein cross: 279 days). In the first month of life, the calf had an episode of respiratory disease and was treated on farm with amoxicillin 7.0 mg/kg and clavulanic acid 1.75 mg/kg–1.75 mg/kg body weight (BW) intramuscular injection once a day for five days (Synulox RTU, 35 mg/mL Amoxicillin trihydrate, 140 mg/mL Clavulanic acid, Zoetis, USA) and meloxicam 0.5 mg/kg BW by subcutaneous injection twice, at an interval of 48 h (Recocam, 20 mg/mL Meloxicam, Bimeda, UK).

**Fig. 1:**
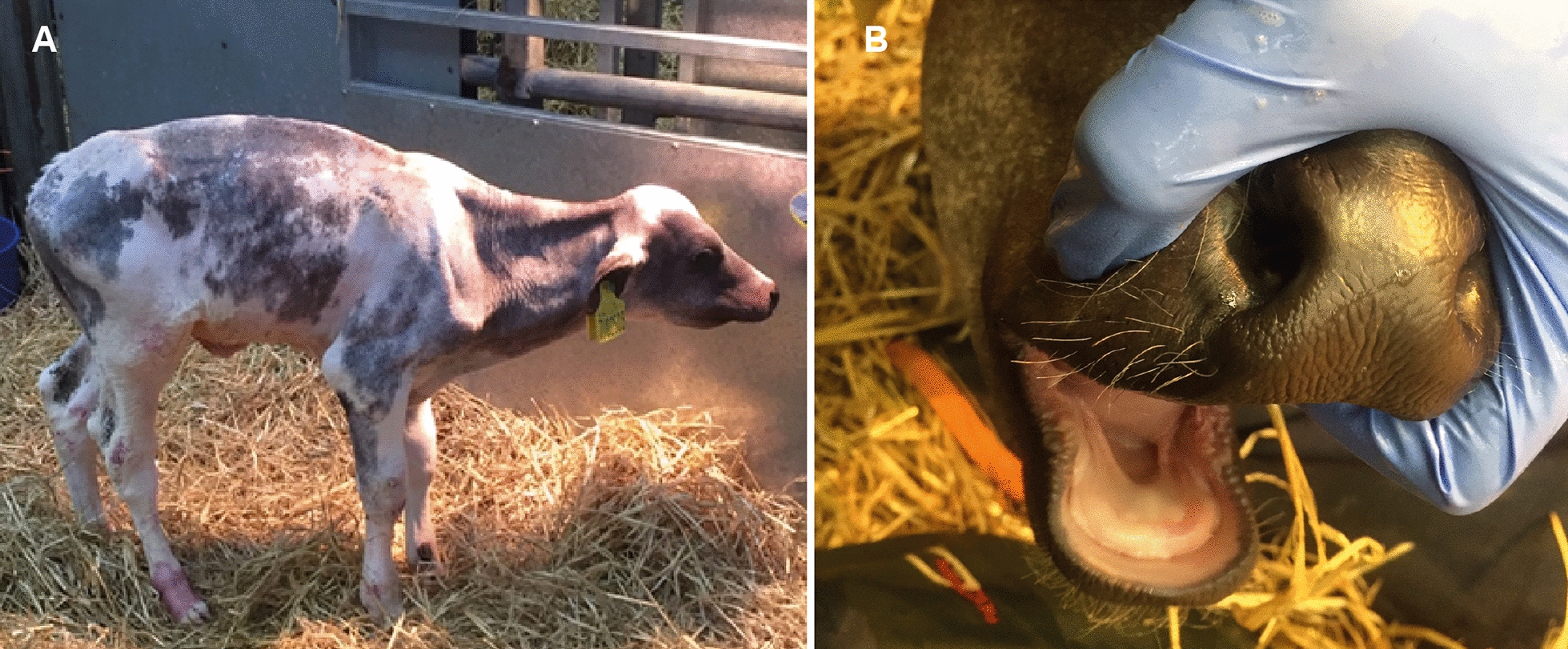
British blue cross calf with hypohidrotic ectodermal dysplasia. **A** Note the short and fine hair all over the body. **B** Note the absence of incisors

On clinical examination at 46 days old, the calf had short, fine hair all over the body (Fig. 1A). In craniocaudal order, the head, neck, thorax, legs and the areas close to the base of the tail were carefully inspected and palpated. The skin of the pinnae had multifocal, slightly shiny appearance, and irregular foci of crusting and/or thickening or flakes of skin were observed. Areas of alopecia were observed on all four legs (predominantly in association with joints) with some showing signs of erosion and ulcerations, likely a result of abrasion (Additional file 1). The extremities were cold to the touch, the body temperature was 37.5 ℃, at the lower end of normal expectation for the conditions, and the respiratory rate was within normal expectations for the conditions at 30 breaths per minute. Some coughing episodes were observed. Bilaterally increased lung sounds (wheezes) were audible on auscultation over the cranioventral thorax. On palpation of the umbilicus, the animal demonstrated signs of discomfort. Hyperextension of the metatarsophalangeal joint, most likely caused by flexor tendon laxity, was also observed.

**Fig. 2:**
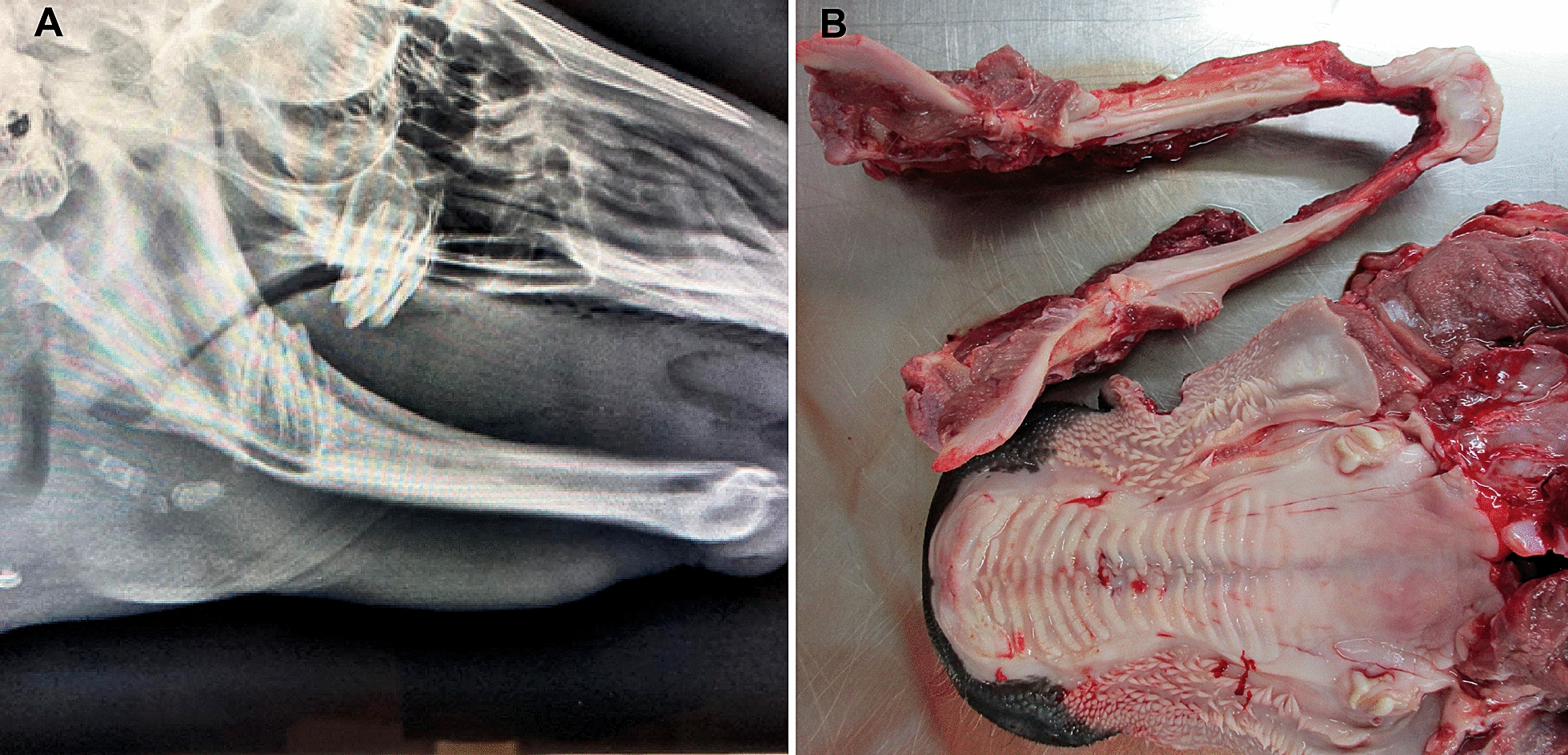
A Skull, radiographic findings of the British blue cross calf with hypohidrotic ectodermal dysplasia. Note the absence of incisors and the presence of only four partially erupted abnormally shaped molars at the caudal aspect of the maxillary and mandibular arcades. **B** Post-mortem dental findings. Note absence of incisors and presence of only one, partially erupted, abnormally shaped cheek tooth at the caudal aspect of each maxillary arcade

In the oral cavity there were no incisors. Only one erupted cheek tooth was palpable at the caudal aspect of each maxillary arcade, and no teeth were palpable on the mandibular arcades (Fig. 1B). Radiographs of the head confirmed the absence of normal permanent dentition, with only one partially erupted, abnormally shaped cheek tooth at the caudal aspect of each maxillary arcade and one unerupted cheek tooth in each mandibular arcade (Fig. 2A).

Ultrasonography of the lungs and abdominal cavity showed wedge-shaped hypoechoic artefacts (from 3 mm to 1 cm) with comet tails on the surfaces of the cranioventral portions of the lung lobes, bilaterally. There was a peri-umbilical area of 3 × 4 cm containing hyperechoic fluid, consistent with an abscess.

A complete blood count (CBC), blood chemistry profile and urinalysis were obtained. Haematological abnormalities included leucocytosis (21.8 × 10^9^; reference range: 4.0–12.0 × 10^9^), neutrophilia (19.0 × 10^9^; reference range: 0.6–4.1 × 10^9^) and lymphopenia (2.2 × 10^9^; reference range: 2.5–7.5 × 10^9^). Hepatic enzymes glutamate dehydrogenase (60.3 U/L; reference range: 0–10 U/L), gamma-glutamyl transferase (53 U/L; reference range: 0–27 U/L) and alkaline phosphatase (505 U/L; reference range: 20–280 U/L) were slightly increased. Urinalysis was unremarkable. A plain blood sample was collected for the BVDV antigen test, which was negative. The calf was placed under a heat lamp and administered oxytetracycline 30 mg/kg BW in two intramuscular injections 72 h apart (Alamacyn LA 300, Oxytetracycline 30 mg/mL, Norbrook, UK) and meloxicam 0.5 mg/kg BW in two subcutaneous injections at an interval of 48 h (Recocam, Meloxicam 20 mg/mL, Bimeda, UK). The calf was fed with two litres of milk replacer twice a day by bottle (AM and PM), containing 125 g of milk powder per litre. In conjunction with husbandry management, the medical treatment improved the animal’s general condition in the following week. Nonetheless, due to the expected difficulties of feeding the animal a solid diet, one month after the admission, when the calf was 76-days old, it was euthanised on welfare grounds.

Hypotrichosis, oligodontia, bronchopneumonia, an umbilical abscess, and a subcutaneous abscess of approximately 1 cm on the medial aspect of the right fore fetlock were all confirmed by gross inspection postmortem. In addition to the complete absence of incisors, only one, partially erupted, abnormally shaped cheek tooth was present at the caudal aspect of each maxillary arcade (Fig. 2B). On sectioning of the mandible at the angle of the ramus bilaterally, one abnormally shaped unerupted tooth was also present at the caudal aspect of each arcade. Lesions typical of chronic bronchopneumonia were found: multifocal fibrous pleural adhesions and pulmonary consolidation cranioventrally with small amounts of mucoid to suppurative material in airways.

Samples from haired skin, nasal planum, and lungs of the affected calf were fixed in 10% neutral buffered formalin, trimmed, processed, embedded in paraffin wax, sectioned at 4 µm, and stained with haematoxylin and eosin (H&E) for histological evaluation. Haired skin samples from three different locations from a normal 4.5-month-old-calf (breed unknown) were used for comparison.

**Fig. 3:**
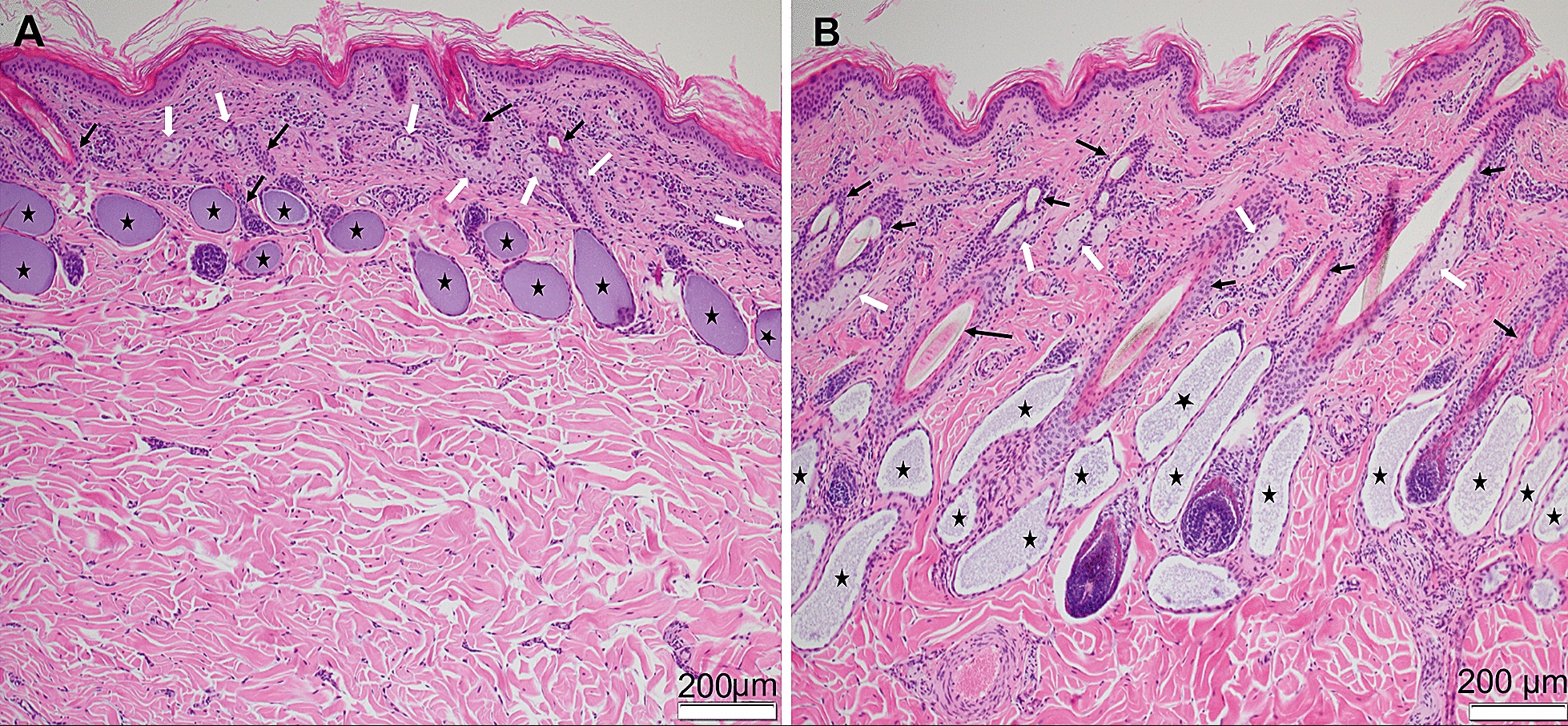
Skin, histological findings. **A** Skin area with marked alopecia from the British blue cross calf with hypohidrotic ectodermal dysplasia. The skin from the affected calf displays a reduced number of hair follicles (black arrows), which also are smaller in size. In addition, there is a decreased number of anagen hairs. Apocrine glands are marked with black stars, and white arrows indicate the sebaceous glands. **B** Skin from a normal 4.5-months-old calf

Histopathological examination of haired skin revealed that in comparison with the control calf, in the skin samples of the affected calf (more pronounced in the biopsy from an area with marked alopecia and mildly in another skin area with erosion), the number of hair follicles was lower, with smaller follicular size and a predominance of telogen-phase hairs (Fig. 3A, B). Apocrine and sebaceous glands were present in all examined skin sections from the affected calf. Although no attempt was made to quantify the density of structures, the apocrine glands appeared to be similar in number and size when compared with the control calf, and the number of sebaceous glands in the affected calf appeared to be similar in sections from both calves. In the area with prominent alopecia, the sebaceous glands seemed smaller in size but they look fully developed in the area with erosion. Multifocal epidermal erosions, and ulceration with attendant mild mixed inflammation and serocellular crust formation and epidermal hyperplasia were also noted. Nasolabial glands and ducts were not observed in the nasal planum sample of the affected calf. Bronchial glands were likewise not clearly visible in the limited number of lung sections examined against a background of inflammation. Marked fibrinous, chronic-active bronchointerstitial pneumonia with multifocal necrotising bronchiolitis, bronchiolar hyperplasia, bronchiolitis obliterans, alveolar multinucleate cells, and patchy type II pneumocyte hyperplasia were noted in samples from the cranial lobes bilaterally.

Based on the clinicopathological findings, the phenotype of the calf was considered consistent with HED.

### Genetic analysis

Genomic DNA was extracted from the EDTA blood sample of the affected calf using the Promega Maxwell RSC DNA system (Promega, Dübendorf, Switzerland). Using the genomic DNA of the affected calf, an individual PCR**-**free fragment library with approximately 400 base pair (bp) inserts was prepared and sequenced for 150 bp paired**-**end reads using the NovaSeq6000 system (Illumina, San Diego, CA, USA). The sequenced reads were mapped to the ARS-UCD1.2 reference genome resulting in an average read depth of approximately 20.2 × [9], and single-nucleotide variants (SNVs) and small indel variants were called. The applied software and steps to process fastq files into binary alignment map (BAM) and genomic variant call format (GVCF) files were in accordance with the 1000 Bull Genomes Project processing guidelines of run 7 [10], except for the trimming, which was performed using fastp [11]. Genomic data was further prepared as previously described [12]. The impact of the called variants was functionally annotated with snpeff v4.3 [13], using the NCBI Annotation Release 106 (https://www.ncbi.nlm.nih.gov/genome/annotation_euk/Bos_taurus/106/; accessed on 17th July 2021), which resulted in the final VCF file, including all individual variants and their functional annotations. In order to find private variants (mutations that are unique to the individual under investigation), the genotypes of the affected calf (publicly available on European Nucleotide Archive under the sample accession number SAMEA8565053) were compared with 5116 cattle genomes from various breeds (> 130 breeds), mostly of run 9 of the 1000 Bull Genomes Project [23] including 576 of the Swiss Comparative Bovine Resequencing project (https://www.ebi.ac.uk/ena/browser/view/PRJEB18113). A total of 86 heterozygous and 8 homozygous private protein-changing single-nucleotide or short indel variants with a moderate or high predicted impact were identified. Afterwards, the identified variants were further investigated for their occurrence in the comprehensive variant catalogue of run 9 of the 1000 Bull Genomes Project [23], including 5116 cattle genomes from various breeds (> 130 breeds), revealed 20 heterozygous and 1 homozygous remaining protein-changing variants exclusively present in the genome of the affected calf and absent in all controls (Additional file 2). The list of remaining variants included no variants in candidate genes for ectodermal dysplasia. A 21,899 bp sized deletion from position 80,516,615 to 80,538,514 on the X chromosome spanning the coding exon 2 of the gene (NM_001081743.2: c.397_502del) was observed, and is predicted to lead to an aberrant protein (NP_001075212.1: p.Met133ValfsTer111) (Fig. 5A, B). This hemizygous gross deletion variant in *EDA* represents a loss-of-function of the encoded ectodysplasin A protein, was absent in any of the cattle genomes used for comparison, and so is considered to be the most likely cause of the observed phenotype.

## Discussion and conclusions

A diagnosis of HED was made based on clinicopathological findings and the presence of a large deletion in *EDA*. In cattle, two main types of HED are described in literature, showing a similar phenotypic expression: most arising from mutations in EDA and one case in EDAR [7] although one other gene–*TSR2*-has been associated with a similar HED phenotypic alteration (hairless streaks) in Pezzata Rossa cattle [14]. However, few types of bovine HED have been characterized to the molecular level [15–17]. HED cases show notable lesions in the integumentary system associated with oligodontia/adontia: homogeneous hypotrichosis, sebaceous glands are reduced in number or morphologically abnormal, and eccrine nasolabial glands are absent in the muzzle. In most of the bovine HED cases, the apocrine sweat glands are absent or reduced in number and may be hypoplastic (hypohidrosis) [16, 18–21]. Together these findings are suggestive of reduced activity of the apocrine sweat glands, resulting in reduced capacity for thermoregulation. A further common finding in HED cases in humans is the absence of cilia (the presence of the cilia was confirmed in the current case in bronchi and bronchioles) and mucous glands in the respiratory tract resulting in susceptibility to respiratory infections [17, 20–22]. Cases of HED in humans and animals that share this phenotype should be suspected of having a defect in the *EDA*, *EDAR* or *EDARADD* gene [8]. Nevertheless, in the case described here, because the abnormality is a very large deletion, filtering for private single-nucleotide variants in *EDA*, *EDAR* and and *EDARADD* genes did not allow the detection of a private substitution or a short indel variant. However, when the genomic data were visually examined for structural variants in three functional candidate genes, an X-linked deletion in the *EDA* gene was identified. It is hypothesized that this deletion occurred de novo or was inherited from a heterozygous dam. Unfortunately, no samples from the parents were available to test these two possibilities.

**Fig. 4:**
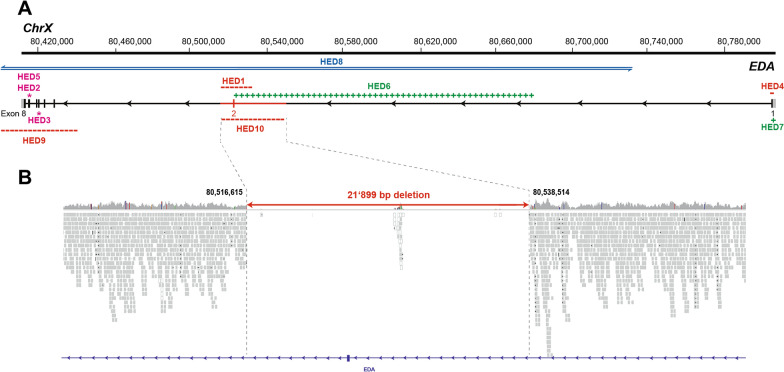
Schematic diagram of the *EDA* gene showing identified pathogenic variant in the British blue crossbred calf and the nine previously reported variants causing hypohidrotic ectodermal dysplasia (HED) in cattle. **A** *EDA* gene structure showing the identified variant location on chromosome X as well as the previously reported pathogenic variants in cattle. Note that the different colours illustrate different kind of variants according to Table 1. **B** Integrative Genomics Viewer screenshot in the region of the 21,899‐bp deletion affecting exon 2 of the *EDA* gene. Note the drop in coverage and the truncated read‐alignments at the deletion breakpoints

The identified pathogenic variant is predicted to affect the coding exon 2 of *EDA,* leading to haploinsufficiency. Recent data from human genome sequencing studies presented in the Genome Aggregation Database (gnomAD) [23] showed that the probability of loss-of-function intolerance score for *EDA* was 0.974, meaning that *EDA* falls within the class of loss-of-function haploinsufficient genes. The *EDA* gene encodes ectodysplasin A which is essential in the ectodysplasin signalling pathway during embryonal and foetal life for normal interaction between the mesoderm and the ectoderm; in particular, it is crucial for the proper formation of hair follicles and tooth buds during development [24]. We assume that the identified pathogenic variant in the *EDA* gene disrupted the encoded protein and caused the observed phenotype similarly to most of the known *EDA*-related forms of HED in cattle (OMIA 000543–9913). A total of nine independent pathogenic X-linked variants of *EDA*, considered responsible for HED, have been previously identified (Table 1; Fig. 4B). Six of them were structural variants [2, 20–22, 25, 26] and three involved a single nucleotide [16, 18, 27]. Our study reports a novel, large deletion in *EDA* and thereby expands the spectrum of causative variants for bovine HED and to emphasize that different loss-of-function mutations in *EDA* cause a homogenous phenotype. For many genes, it is known that the nature of the genetic alteration influences the phenotypic outcome—that is, the severity of a congenital defect varies or differs entirely according to the exact nature of the variant. For example, myostatin (*MSTN*) controls prenatal muscular development and there are many genetic variants known in cattle (OMIA 000683–9913) with highly divergent phenotypes, ranging from the extreme double muscling of the 11-bp deletion common in the Belgian Blue breed to a more subtle muscular over-development with the missense variant that is common in the Limousin breed [28]. For *EDA* this seems not to be the case in cattle, as both structural and single nucleotide variants obviously cause a broadly similar phenotype, which is critical for diagnostic clinicians and pathologists.

**Table 1:** Summary of known causal variants of *EDA* causing hypohidrotic ectodermal dysplasia (HED) in cattle (based on OMIA 000543–9913), with summary of breed and phenotype

Allele	Type of variant	EDA variant details (reference: gDNA: NC_037357.1; cDNA: NM_001081743.2; protein: NP_001075212.1)	Phenotype and breed	References
HED1	160 kb deletion	g.unknown precise breakpoints c.397_502del p.Met133ValfsTer111	Hypotrichosis and oligodontia, but phenotype not described in detail and no histology presented Holstein	[2]
HED2	Splice site variant	g.80411671A > C c.924+2T > G p.unknown effect on transcription	Hypotrichosis and oligodontia (absence of incisors: presence of only one molar on each side of the upper jaw). Report noted generalized hypotrichosis, thin dermis with sparse, atrophic hair follicles and reduced density of sweat glands, dry muzzle, and absence of eccrine nasolabial, tracheal, and bronchial glands Holstein	[18]
HED3	Nonsense (stop-gain)	g.80415626G > A c.730C > T p.Arg244*	Hypotrichosis and oligondontia (absence of incisors, presence of only 4 maxillary premolars, and a single mandibular premolar, for a total of 5 teeth). Skin histology: On histopathologic examination, very few normal anagen hair follicles were present and in the small, scattered foci of all biopsies, there was total underdevelopment of hair follicles. Hypoplastic follicles were common, contained smaller than normal hair shafts and fewer than normal adnexal glands (both sebaceous and epitrichial) Red Angus-Charolais-Simmental cross	[16]
HED4	19 bp deletion	g.80803015_80803033del c.48_66del p.Ala16Ser22fsTer55	Hypotrichosis and oligodontia (absence of incisors: presence of only one molar on each side of the upper jaw). Skin histology: The hair follicles and adnexal glands of the affected animal were smaller than normal Holstein	[25]
HED5	Splice site variant	g.80411795C > A c.802C > A p.unknown effect on transcription	Hypotrichosis and complete hypodontia. Skin histology not reported Holstein Friesian	[27]
HED6	161 bp insertion	Not reported	Hypotrichosis and oligodontia (absence of incisors and only two erupted premolar teeth). Skin: hypotrichosis or hairlessness due to a reduction in hair follicle complexes, mild superficial perivascular dermatitis involving the superficial and middle vascular plexuses and characterized by mixed mononuclear and polymorphonuclear cellular infiltrates, mild irregular epidermal hyperplasia and dilation of some lymph vessels was also found. The sweat glands were dilated with a flattened, inactive luminal epithelium. The muzzle was without nasolabial glands. Glandular structures were not observed in the trachea and bronchi Holstein	[21]
HED7	4 bp insertion	g.80802800_80802801insCCCT c.280_281insAGGG p.Gly94GlnfsTer49	Hypotrichosis and hypodontia. This is a brief report, and the description of the phenotype is restricted to reference to hairlessness on most of the body, except for the distal parts of the extremities, with severe hypothermia and skin abrasions over the carpal and tarsal joints Japanese Black	[26]
HED8	3.76 Mb inversion	g.7174882_80737442inv p.unknown effect on transcription	Hypotrichosis and oligodontia (absence of incisors, one malformed premolar on each side of the upper jaw). Reduced size of sweat glands, sebaceous glands, and hair follicles in the affected animals. Increased density but reduced size of sebaceous glands and pilary canals Holstein	[20]
HED9	53 kb deletion	g.80382423_80435202del p.unknown effect on transcription	Hypotrichosis, anodontia/oligodontia, and absent or defective ectodermally derived glands. Histologically, mostly small calibre, hair follicles and larger follicles with medullated hairs were only present on the chin, tail, eyelids, tragus and distal limbs Red Angus × Simmental	[22]
HED10	22 kb deletion	g.80516615_80538514del c.397_502del p.Met133ValfsTer111	British blue × Holstein–Friesian	This study

## Supplementary Information

**Additional file 1.** British blue cross calf with hypohidrotic ectodermal dysplasia. A. Note the areas of erosion and the variation in degree of alopecia on the thorax (**A**), over the carpi (**B**), and over the stifle joint (**C**).

**Additional file 2. **List of 21 remaining protein-changing variants with a predicted moderate or high impact only present in the affected calf after the comparison to the global control cohort of 5116 genomes of other breeds.

## Data Availability

Whole-genome sequence data generated from the affected calf is available under study accession PRJEB28191 and sample accession SAMEA8565053 from the European Nucleotide Archive (ENA).

## Abbreviations

BAM: Binary alignment map
bp: Base pair
BVDV: Bovine viral diarrhoea virus
EDTA: Ethylenediaminetetraacetic acid
IGV: Integrative Genomics Viewer
OMIA: Online Mendelian Inheritance in Animals, https://omia.org/home/
SNV: Single-nucleotide variant
WGS: Whole-genome sequencing
