# Supplementary material for: A large deletion encompassing exon 2 of the ectodysplasin A (EDA) gene in a British blue crossbred calf with hypohidrotic ectodermal dysplasia
Source: Acta Vet Scand. 2022 Sep 6;64:23. doi: 10.1186/s13028-022-00641-2 (PMC9446731; doi:10.1186/s13028-022-00641-2)
Supplement: Supplementary file 1 — Additional file 1. British blue cross calf with hypohidrotic ectodermal dysplasia. A. Note the areas of erosion and the variation in degree of alopecia on the thorax (A), over the carpi (B), and over the stifle joint (C). [file 13028_2022_641_MOESM1_ESM.docx]

**Additional File 2:** British blue cross calf with hypohidrotic ectodermal dysplasia. A. Note the areas of erosion and the variation in degree of alopecia on the thorax (A), over the carpi (B), and over the stifle joint (C).


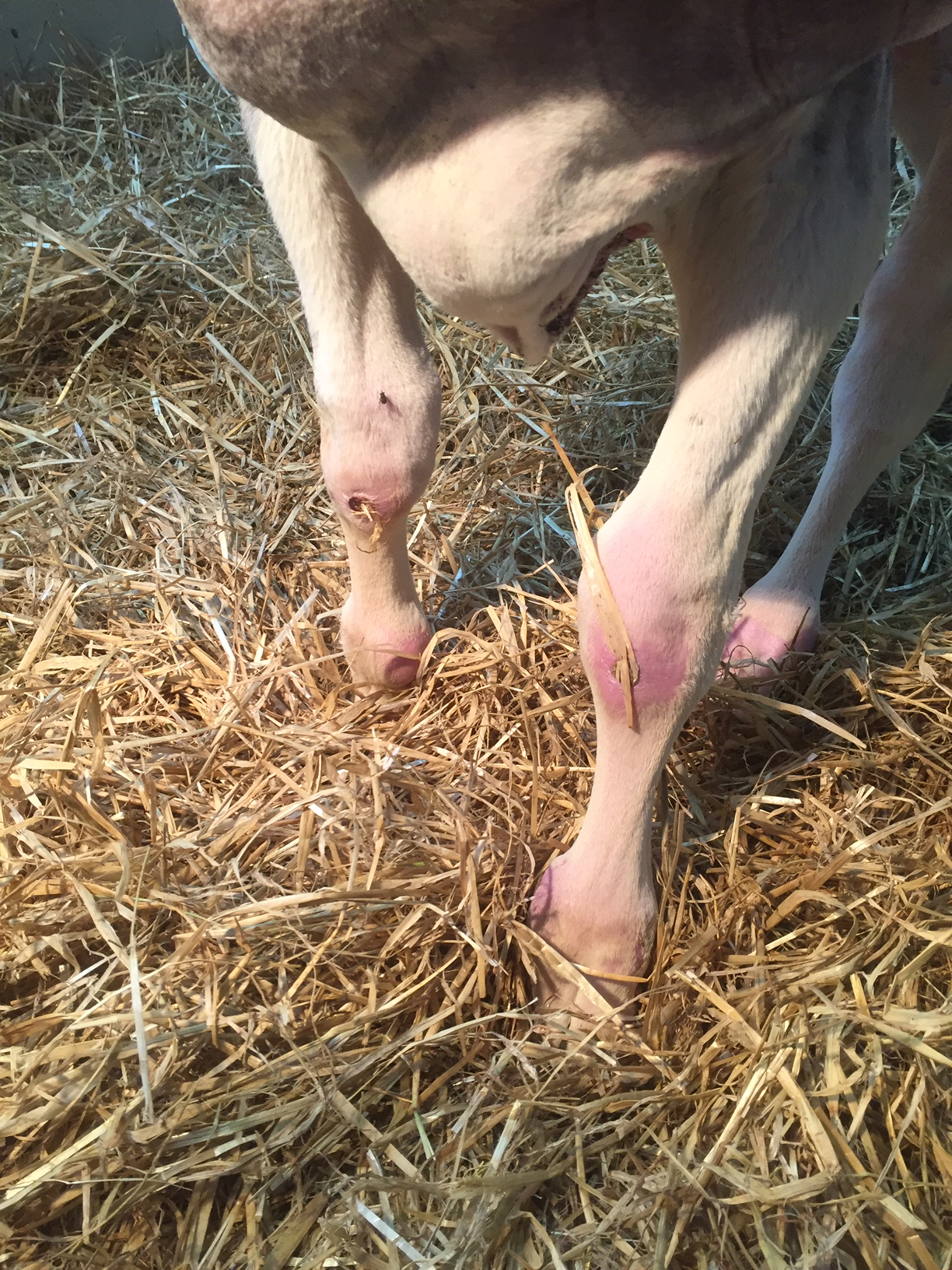

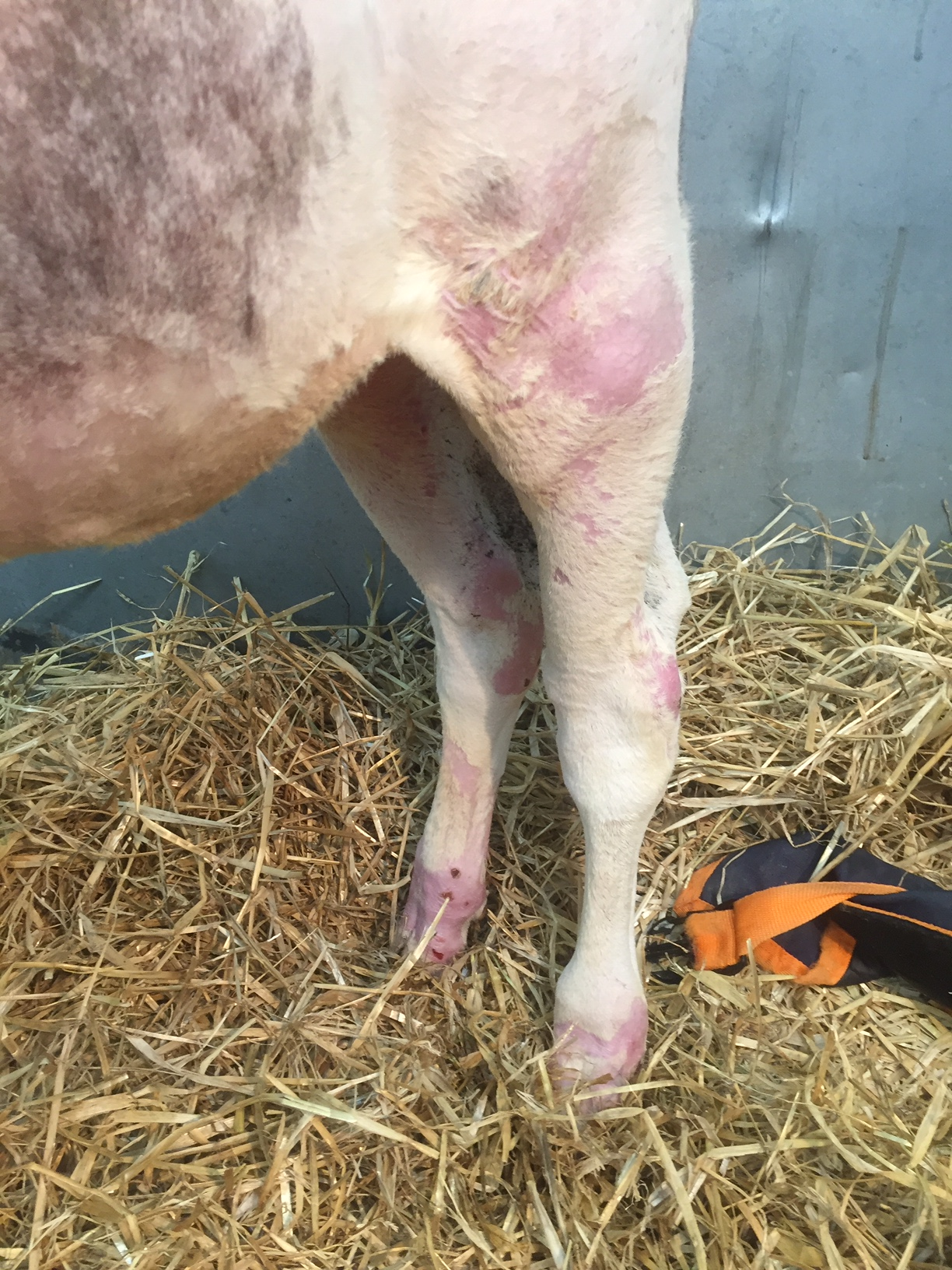

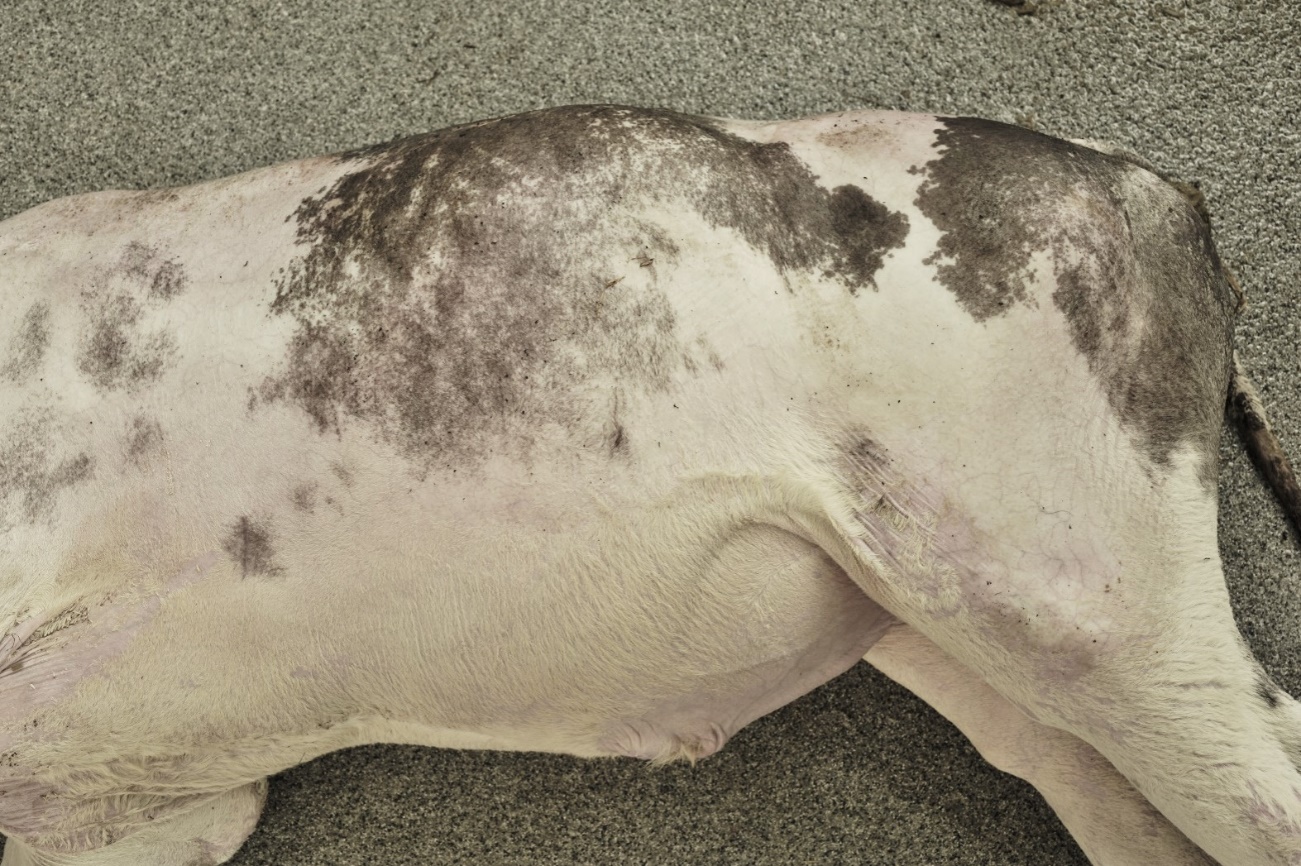


B

C

A
